# Gene Co-Expression Network Analysis Reveals Key Regulatory Genes in *Metisa plana* Hormone Pathways

**DOI:** 10.3390/insects14060503

**Published:** 2023-05-30

**Authors:** Vinothienii Vengatharajuloo, Hoe-Han Goh, Maizom Hassan, Nisha Govender, Suhaila Sulaiman, Nor Afiqah-Aleng, Sarahani Harun, Zeti-Azura Mohamed-Hussein

**Affiliations:** 1 Institute of Systems Biology, Universiti Kebangsaan Malaysia, Bangi 43600, Selangor, Malaysia; p112032@siswa.ukm.edu.my (V.V.); gohhh@ukm.edu.my (H.-H.G.); maizom@ukm.edu.my (M.H.); nishag@ukm.edu.my (N.G.); zeti.hussein@ukm.edu.my (Z.-A.M.-H.); 2 FGV R&D Sdn Bhd, FGV Innovation Center, PT23417 Lengkuk Teknologi, Bandar Baru Enstek, Nilai 71760, Negeri Sembilan, Malaysia; suhaila.s@fgvholdings.com; 3 Institute of Marine Biotechnology, Universiti Malaysia Terengganu, Kuala Nerus 21030, Terengganu, Malaysia; afiqahaleng@umt.edu.my; 4 Department of Applied Physics, Faculty of Science and Technology, Universiti Kebangsaan Malaysia, Bangi 43600, Selangor, Malaysia

**Keywords:** *Metisa plana*, WGCNA, functional enrichment analysis, protein–protein interaction, network clustering, pathway analysis, RNA-interference

## Abstract

**Simple Summary:**

The increasingly growing demand for palm oil (PO) blankets heavy pressure for sustainable PO production. PO, the world’s most efficient oilseed crop, is being used in various applications, including food, cosmetics, and biofuels. However oil palm is always at risk being exposed to *Metisa plana* infestation, which contributes to the negative impacts on the oil palm plantations and industries. Therefore, conventional pesticides are routinely applied as a control measure in outbreak management. Nevertheless, the perpetual occurrences of *M. plana* infestation remain, affecting the overall yield productivity. This study uses an integrated bioinformatics approach, including a gene co-expression network and clustering method, to screen, identify, and determine the key genes involved in controlling insect hormone biosynthesis with ecdysone and juvenile hormones among them. The findings provide key regulatory genes (*Hnf4*, *Hr4*, *MED14*, *Usp*, *Tai*, and *Trr*) that could be used as potential targets for gene silencing technologies such as RNA interference (RNAi) desired in understanding *M. plana* gene function.

**Abstract:**

*Metisa plana* Walker (Lepidoptera: Psychidae) is a major oil palm pest species distributed across Southeast Asia. *M. plana* outbreaks are regarded as serious ongoing threats to the oil palm industry due to their ability to significantly reduce fruit yield and subsequent productivity. Currently, conventional pesticide overuses may harm non-target organisms and severely pollute the environment. This study aims to identify key regulatory genes involved in hormone pathways during the third instar larvae stage of *M. plana* gene co-expression network analysis. A weighted gene co-expression network analysis (WGCNA) was conducted on the *M. plana* transcriptomes to construct a gene co-expression network. The transcriptome datasets were obtained from different development stages of *M. plana*, i.e., egg, third instar larvae, pupa, and adult. The network was clustered using the DPClusO algorithm and validated using Fisher’s exact test and receiver operating characteristic (ROC) analysis. The clustering analysis was performed on the network and 20 potential regulatory genes (such as *MTA1-like*, *Nub*, *Grn*, and *Usp*) were identified from ten top-most significant clusters. Pathway enrichment analysis was performed to identify hormone signalling pathways and these pathways were identified, i.e., hormone-mediated signalling, steroid hormone-mediated signalling, and intracellular steroid hormone receptor signalling as well as six regulatory genes *Hnf4*, *Hr4*, *MED14*, *Usp*, *Tai*, and *Trr*. These key regulatory genes have a potential as important targets in future upstream applications and validation studies in the development of biorational pesticides against *M. plana* and the RNA interference (RNAi) gene silencing method.

## 1. Introduction

Palm oil, derived from the oil palm (*Elaeis guineensis* Jacq.) mesocarp, accounts for approximately 40% of globally traded vegetable oils. Oil palm is the most important and efficient oil-seed crop worldwide due to its exceptionally low land footprints [1]. The perennial crop has an average life cycle of more than 25 years. Since the beginning of the 21st century, the demand for palm oil has increased tremendously and over 60% of the palms and fats are exported globally [2,3]. Malaysia is the second-largest palm oil producer after Indonesia [3,4] and is ranked amongst the largest palm oil exporters [3,5]. Both countries contribute up to 80.5% of global palm oil production [6].

Oil palm plantations are highly susceptible to insect pest infestations such as bagworms (*Metisa plana* Walker and *Pteroma pendula* Joannis), nettle caterpillars (*Setora nitens*), and rhinoceros beetle (*Oryctes* spp.) [7]. Amongst them, *Metisa plana* Walker (Lepidoptera: Psychidae) has emerged as a serious yield-impeding concern of the recent time [8,9]. The *M. plana* infestation reduces the production of fruit bunches which directly impacts the quantity and quality of the oil production [9]. According to Halim et al. (2017), *M. plana* infestation reduces up to 57% of oil palm productivity [10]. Referring to recent statistical evidence indicated in Malaysia, *M. plana* infestation attributed to a significant decrement in crude palm oil production from 19.14 million metric tonnes in 2020 to 18.11 million metric tonnes, in 2021 [11]. Moreover, *M. plana* infestation has established a long history with perpetual occurrence reported for over the last five decades [8]. Despite numerous control measures (selective applications of chemical and biological controls) the natural occurrence of *M. plana* outbreak in oil palm plantations remains uncontrollable [12,13]. Due to its highly notorious and invasive nature, *M. plana* has been declared one of the most dangerous insect pests under the Plant Quarantine Act 1976 (No. 167) [14].

*M. plana*, is a holometabolous (complete metamorphosis) insect with four distinct developmental stages. It begins its life cycle as an egg, which then develops into instar larvae and pupa, and finally matures into adulthood. The life cycle of *M. plana* ranges between 80–113 days. In larvae, the first to the third instars are considered the most critical phases throughout the lifecycle. During the larval phase, *M. plana* actively feed on oil palm leaves and camouflage their silken cases in small pieces of leaves [15,16]. The larval growth and pupal development stage are regulated by ecdysone and juvenile hormones. These hormones control the moulting, metamorphic transition duration, and growth duration. The activity and effects of hormones in *M. plana* are influenced by a variety of regulatory genes and enzymes. These genes and enzymes are crucial for regulating the biosynthesis pathways of insect hormones and ensuring the expression of downstream functional genes. In essence, they play a critical role in controlling the action of hormones in *M. plana* [17].

Regulatory genes modulate the expression or transcription rate of one or more structural genes [18]. They are essential in the transcriptional regulation of functional genes and are involved in regulating a diverse range of physiologically important processes in insects, such as ecdysone titers, that control the pulses of the ecdysone during the development of insects [19,20,21]. In *Drosophila melanogaster*, several regulatory genes such as *Sean*, *Ouib*, and *MId* specifically control the regulation of Halloween genes which encode a series of ecdysone biosynthetic enzymes involved in facilitating steroidogenesis. These regulatory genes control the transcription of both *Nvd* and *Spok* of the Halloween genes which are responsible for the synthesis and degradation of ecdysone [19]. In *Bombyx mori*, the degradation of juvenile hormone (JH) is regulated by the *BmFOXO* regulatory gene through the action of JH-degrading enzyme-encoding genes such as *JHE*, *JHEH*, and *JHDK* [22].

To gain a better understanding of the molecular and biological processes involved in the growth and development of *M. plana*, various studies have employed omics technologies as part of an integrated pest management (IPM) approach. These studies aim to uncover the underlying mechanisms that contribute to *M. plana*’s growth and development. For example, transcriptomics approaches have been applied to identify chitin biosynthesis regulatory genes controlling the moulting in larvae and pupae of *M. plana* [23] and detoxification genes such as *P450*, *GST*, and *CCE* [24]. In parallel, this study aims to construct a weighted gene co-expression network from *M. plana* transcriptome data and identify key regulatory genes involved in the hormone biosynthesis of *M. plana* third instar larvae.

Weighted gene co-expression network analysis (WGCNA) is a computational approach used in systems biology to establish a scale-free network of related modules based on correlation algorithm and groupings of similar expressions [25]. In this study, WGCNA and graph clustering techniques were combined to identify regulatory genes that control the hormone biosynthesis pathways in *M. plana* [26,27]. Several studies highlighted the application of gene clustering into functional modules using the WGCNA approach in elucidating regulatory mechanisms: embryogenesis in the hemipteran insect *Nilaparvata lugens* for understanding insect patterning and evolution [28] and wax biosynthesis in *Ericerus pela* Chavannes [29]. These studies demonstrated that the knowledge obtained from the identified key modules, pathways, and genes can be used to better understand the biological mechanisms.

The long-term goal of this study was to identify *M. plana* regulatory genes that can be applied in RNAi technology, which has become a promising tool in pest management and has been increasingly used in the integrated pest management approaches [30]. RNA interference (RNAi) is a natural mechanism that cells use to regulate gene expression by targeting and degrading specific RNA molecules. RNAi technology has been widely used in pest control by targeting essential insect genes, leading to reduced pest survival and reproduction [31]. Since RNAi is a sequence-specific method of suppressing a targeted gene expression [32,33], it can be used as an effective control measure for *M. plana* infestation. With the discovery of *M. plana* regulatory genes obtained from the WGCNA, these genes may serve as candidate targets for RNAi technology in future applications.

Several studies used this approach to identify target genes for RNAi in various pests. For example, a study by Zhu et al. (2011) used WGCNA to identify genes involved in the moulting process of the cotton bollworm and then targeted these genes with RNAi, resulting in reduced survival and development of the insects [34]. Another study by Zhang et al. (2021) used combined transcriptomic analysis and RNAi technology to study the effects of methoxyfenozide on the ecdysone signalling pathway of *Spodoptera exigua* [35]. On that account, applying WGCNA is essential for RNAi technology, as it is a powerful tool for identifying and targeting essential genes in insect pests, which leads to potential applications in pest control.

## 2. Materials and Methods

### 2.1. Data Pre-Processing

A total of 193,686 genes from nine transcriptome datasets of different *M. plana* developmental stages, as reported previously [23], were used in the weighted gene co-expression network analysis (WGCNA). The DESeq2 package was used to remove lowly expressed genes defined as genes <ten read counts, as identified in the count (gene expression) dataset. The filtered count data was subjected to normalisation using the Variance Stabilising Transformation (VST) built-in function in DESeq2 package from Bioconductor (http://www.bioconductor.org) (accessed on 26 August 2021). The VST method adopts a logarithmic transformation otherwise known as regularised log transformation (rlog) to shrink the differences between genes with high and low expression counts. This step reduces the impact of outliers and stabilises variance across the broad range of expression values. The normalisation process starts with the estimation of size factors, which was then used to normalise the count number for each gene. Next, the regularised log transformation was applied to the normalised count numbers, resulting into a matrix of transformed values. This transformation ensures that the variance of each gene is approximately constant across the expression value range. To perform WGCNA, the genes with high variance, determined by the q95 percentile, were selected. This selection criterion is based on the assumption that genes with the highest variance are more likely to be biologically relevant and informative for understanding the underlying regulatory mechanism [36]. R version 4.1.2 software (R Foundation for Statistical Computing, Vienna, Austria) was used to perform the analyses (accessed on 25 August 2021).

### 2.2. Construction of Weighted Gene Co-Expression Network Analysis (WGCNA)

A weighted gene co-expression network was constructed using the WGCNA package, an R library [25]. The WGCNA was performed on the normalised *M. plana* gene expression data. The hierarchical clustering (hclust) function was applied to detect the presence/absence of outlier samples. Soft-thresholding power, β, was set based on the ‘pickSoftThreshold’ function output. At R^2^ ≥ 0.80, a scale-free network was constructed. WGCNA was constructed using the blockwiseModules function. A weighted adjacency matrix was generated using the adjacency function, after which the matrix was converted into Topological Overlap Matrix (TOM) using the *TOMsimilarity* function. Finally, TOM measures between pairs of genes under all possible combinations were used as input in the average linkage hierarchical clustering, dissimilarity matrix (dissTOM = 1 − TOM). Next, the modules were identified using the dynamic tree cut algorithm with the following parameters: β = 12, networkType = “signed”, minModuleSize = 30, and mergeCutHeight = 0.25.

### 2.3. Key Modules Selection and Hub Genes Identification

The module eigengene (ME) was characterised by the first principal component that reflects the expression level of each module [25]. The relationship between module eigengene and the developmental stages of *M. plana* was estimated using Pearson’s correlation values. The heatmap package from R library was used to visualise the correlations between the module and *M. plana*’s developmental stage. ME with a significant positive correlation with *M. plana* developmental stage was selected for further analysis. The hub genes or genes with the highest degree of connectivity were identified based on the estimation of signedKME or module membership (MM) function in the WGCNA package. Module membership (kME) estimates the correlation between the gene expression profile and the module eigengene for each gene in the module. Genes with kME ≥ 0.80 are defined as hub genes.

### 2.4. Functional Annotation of Unannotated Genes

The following corresponding gene information of each module was extracted: geneID, BLAST description, HMMER description, and gene symbols were fetched. This information was used to prepare the gene list containing gene symbols or synonyms used in the enrichment analysis. Genes without annotation or those characterised as hypothetical proteins were reannotated using Annotation Retrieval of Gene Ontology Terms (ARGOT) version 2.5 (http://www.medcomp.medicina.unipd.it/Argot2) (accessed on 4 July 2022), a web-based gene function prediction tool. ARGOT annotates the function based on the Gene Ontology (GO) annotation of hits retrieved from BLAST and HMMER searches. In combination with a weighting scheme and clustering approach, the most accurate GO terms were selected to annotate the target proteins [37].

### 2.5. Functional Enrichment Analysis of the Key Modules

Metascape v3.5 (http://metascape.org) (accessed on 23 August 2022) database was used in the GO enrichment analysis to infer the biological information of the key modules [38]. The functional enrichment analysis was performed using the following default parameters: *p*-value < 0.05, minimum count set at 3, and enrichment factor > 1.5 for clusters with membership similarities of Kappa scores > 0.3 [38]. The most statistically significant term within a cluster was chosen to represent the biological role of the cluster per se. Protein–protein interaction (PPI) enrichment analysis was conducted on hub genes using the Metascape tool. Cytoscape software version 3.9.0 [39] was used to visualise the PPI network.

### 2.6. Network Clustering Analysis

DPClusO algorithm generates clusters of densely connected regions from a gene network. The overlapping clusters are described with several biological processes related to the hormone biosynthesis pathway. DPClusO constructs an undirected graph consisting of a finite set of nodes, *N* and a finite set of edges, *E*. Two key parameters, namely the density, *d_k_* and cluster property, *cp_nk_*, are applied in the DPClusO algorithm of the cluster network analysis [40,41]. The default *cp* value, 0.5 was used with reference to the previous studies [41,42,43]. The cluster property of node *n* with respect to cluster *k* is represented by the following equation:

(1) $${cp}_{nk}=\frac{\left|E_{nk}\right|}{d_{k}\times \left|N_{k}\right|}$$

*N_k_* refers to the number of nodes in cluster *k*. *E_nk_* is the total number of edges connecting the node *n* with nodes of cluster *k*.

### 2.7. Fisher’s Exact Test Analysis

Fisher’s exact test was used in the enrichment of regulatory gene clusters. The statistical test analysed the regulatory and non-regulatory genes in a contingency table format as shown in (Table 1) [41,44,45]; a, b, c, and d each denote independent gene numbers. The best set of clusters was identified by calculating the average significance of all given clusters per se. Fisher’s exact test *p*-values were used to assess the regulatory gene enrichment in each cluster.

### 2.8. SScore and ROC Statistical Analysis

A significant score (SScore) is defined as −log (*p*-value) and it estimates the prediction confidence of potential regulatory genes for each gene based on the *p*-value of the clusters. The lowest *p*-value of a gene was used to measure the SScore. A gene can belong to multiple clusters and equate to more than one *p*-value. The potential regulatory genes are identified by estimating the power of SScore [41,46]. The evaluation of the True Positive Rate (*TPR*) and False Positive Rate (*FPR*) was calculated using a series of threshold (th) SScore. The fraction of true positive predictions in all positive data is *TPR*, and the fraction of false positive predictions in all negative data is *FPR*. The following equations were used to calculate *TPR* and *FPR*:

(2) $$TPR=\frac{TP}{TP+FN}$$

(3) $$FPR=\frac{FP}{FP+TN}$$

Based on the listed equations above, true positive (*TP*), false positive (*FP*), true negative (*TN*), and false negative (*FN*) were known as the number of regulatory genes with SScore ≥ *th*, number of non-regulatory genes with SScore ≥ *th*, number of non-regulatory genes having SScore < *th*, and number of regulatory genes having SScore < *th*, respectively. Finally, the Area Under the ROC Curve (AUC) test was used to evaluate the efficiency of SScore in identifying potential regulatory genes. ROCR package, an R library, was used to estimate AUC [47].

### 2.9. Pathway Enrichment Analysis

**Figure 1:**
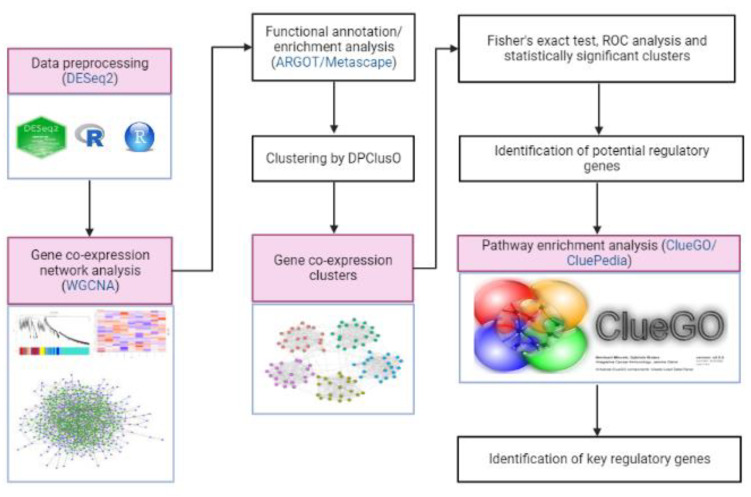
A step-by-step procedure in identifying the key regulatory genes that are involved in the hormone-mediated pathways of *M. plana* third instar larvae.

ClueGO/CluePedia, a Cytoscape plug-in tool, was used in the pathway enrichment analysis to obtain the biological roles of clusters with potential regulatory genes and gene clusters [41,48]. The false discovery rate (FDR) of each pathway was calculated by ClueGO/CluePedia using a hypergeometric test with Bonferroni correction to determine its significance. The method flowchart is shown in Figure 1.

## 3. Results

### 3.1. Data Processing

After the pre-filtration process, a total of 130,020 transcripts were obtained from the 193,686 transcripts, where 67.12% of the transcripts were normalised and 6501 genes exhibiting the top q95 percentile of high expressional variance were selected for the WGCNA analysis.

### 3.2. Weighted Gene Co-Expression Network Construction and Key Modules Selection

**Figure 2:**
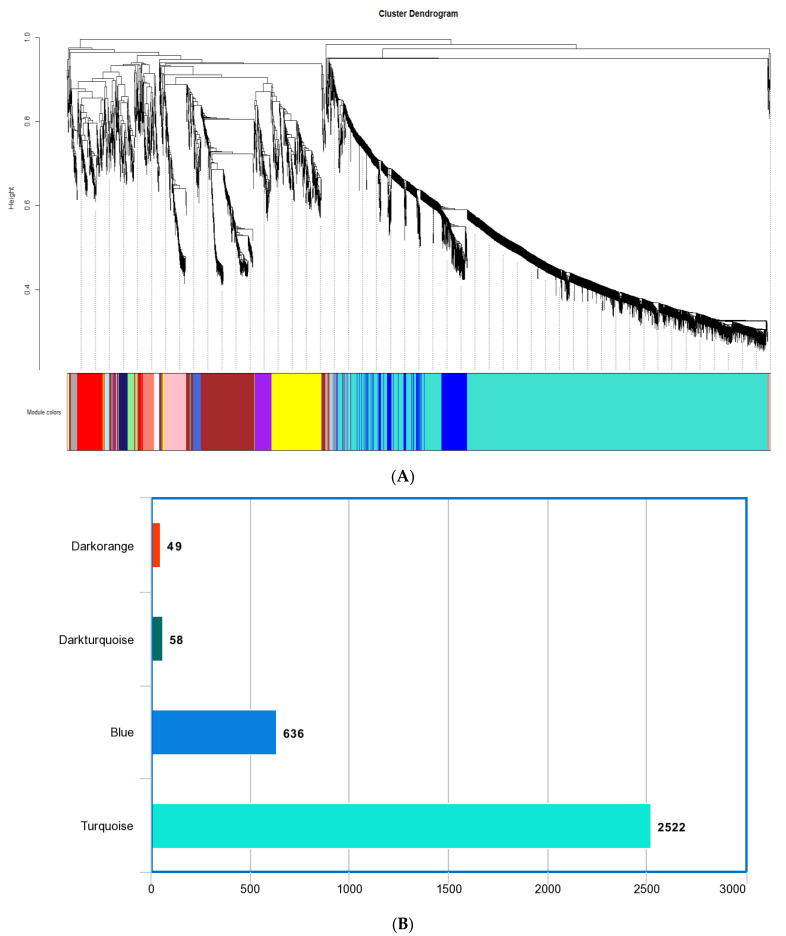
The gene co-expression network analysis of M. plana. (**A**) Dendrogram of genes clustered based on a dissimilarity measure (1−TOM). Each colour represents a module, and each branch represents the genes. There are 34 co-expressed modules. (**B**) The bar chart represents the number of genes in four key modules that are related to the third.

The *M. plana* gene co-expression network was constructed from 6501 genes obtained from different developmental stages: the egg, third instar larvae, pupa, and adult. The genes were grouped into 34 co-expression modules using the average linkage hierarchical clustering algorithm (Figure 2A). The size of all co-expression modules ranges from 30 to 2522 genes. Since the grey module could not be annotated successfully, the module was excluded from further analysis. For further analysis, we selected the following modules that corresponded to the third instar larvae of *M. plana*: turquoise, blue, dark-turquoise, and dark-orange modules (as shown in Figure 2B). These modules specifically represented the stage of third instar larvae in *M. plana* and were chosen for subsequent analysis. The descriptive information on the *M. plana* third instar larvae modules is tabulated in Table 2. Further information on the modules generated from gene co-expression network analysis is provided in the supplementary materials (Figures S1–S4, Tables S1 and S2).

### 3.3. Functional Annotation

Prior to the functional enrichment analysis, a gene list was prepared, and gene information was extracted from an annotated *M. plana* transcriptome dataset, as obtained from a previous study [23]. A total of 2590 genes were extracted from four modules associated with third instar larvae. A total of unannotated 1092 genes were characterised as hypothetical proteins based on the findings from previous studies [23]. ARGOT tool was used to facilitate the annotation of these genes based on Gene Ontology terms. Genes with no annotations were removed. The annotated genes are categorised into various classes of enzymes, transporters, binding proteins, metabolic processes, catalytic activity, and kinase activity. The descriptive statistic of the functional annotation is provided in Table 3.

### 3.4. Functional Enrichment Analysis of Key Modules

The selected hub genes were functionally enriched to obtain their biological information towards understanding the mechanism involved in *M. plana*, and they were enriched biologically. Therefore, the extracted genes from all four modules were pooled into a single unified gene list and fed into the Metascape tool. The biological processes assigned to the hub genes are listed as follows in Table 4, with the top-most 20 clusters with enriched representative terms.

### 3.5. Protein–Protein Interaction Network Analysis

**Figure 3:**
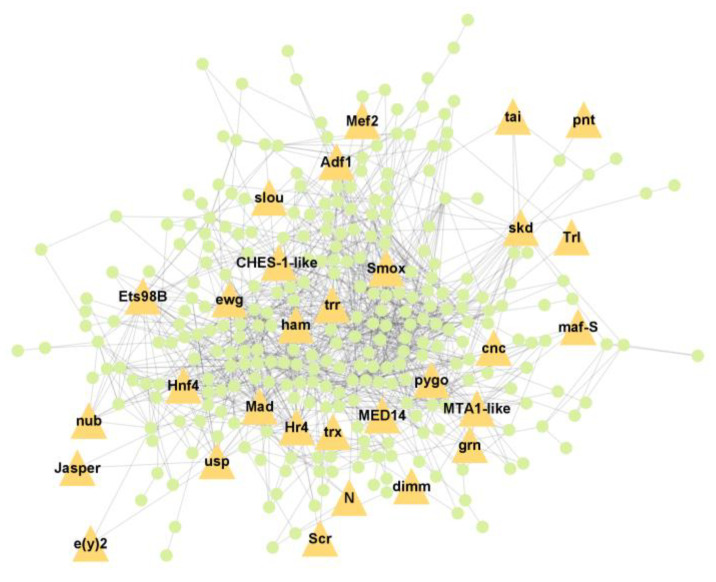
Protein–Protein interaction network of the hub genes. The yellow triangle nodes represent the regulatory genes in the network, and the light green nodes represent the non-regulatory genes.

PPI analysis was performed on the hub genes to analyse the connections between the proteins. The PPI network was constructed with 338 nodes and 1167 edges. The circular and triangular nodes represent 308 non-regulatory genes and 30 regulatory genes, respectively (Figure 3).

### 3.6. Network Clustering Analysis and Pathway Enrichment Analysis

The highly interconnected region with similar biological processes clustered by DPClusO showed five clusters with density values of 0.5, 0.6, 0.7, 0.8, and 0.9 and a cp value of 0.5 (Table 5); smaller density values resulted in greater cluster sizes and fewer number of clusters. ROC analysis was performed to screen the suitable density value clusters for the pathway enrichment analysis.

**Figure 4:**
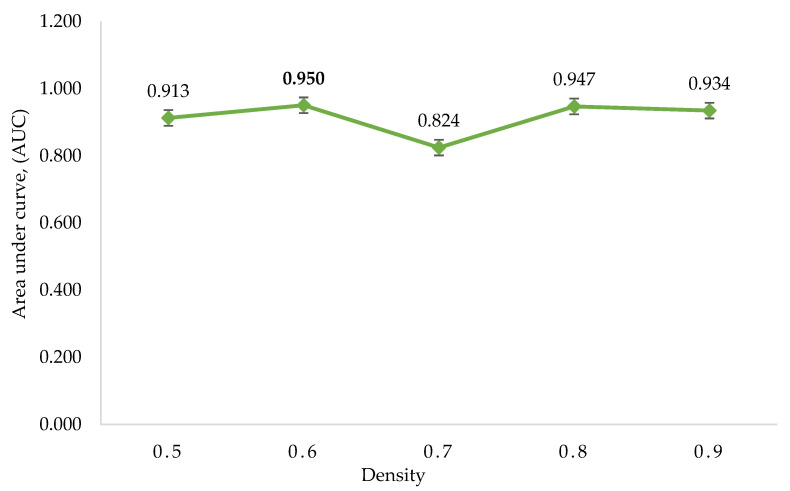
The area under the curve (AUC) was calculated using DPClusO. The density value of 0.6 has the highest AUC, followed by clusters generated from density values of 0.8, 0.9, 0.5, and 0.7.

The *p*-values of Fisher’s exact test were used to assess the regulatory gene enrichment in the identified clusters, followed by the assignment of SScore on each gene based on the clusters’ *p*-value. Five ROC curves were created by utilising the SScore corresponding to the five sets of clusters. The AUC of five ROC curves is shown in (Figure 4, Table 6). The maximum AUC was 0.95, generated from the density value of 0.6. Finally, the regulatory genes found within the statistically significant clusters of the set corresponding to density 0.6 were selected as potential regulatory genes.

A total of 227 significant clusters with a density value of 0.6 (*p*-value < 0.05) were identified. The critical regulatory genes found within the statistically significant cluster were considered significant and thus, analysed further. Table 7 shows a list of potential regulatory genes identified from the highly significant clusters, i.e., Cluster 3, Cluster 7, Cluster 8, Cluster 9, Cluster 11, Cluster 12, Cluster 14, Cluster 24, Cluster 47, and Cluster 53. Genes in the dark green octagon nodes represent potential regulatory genes.

**Figure 5:**
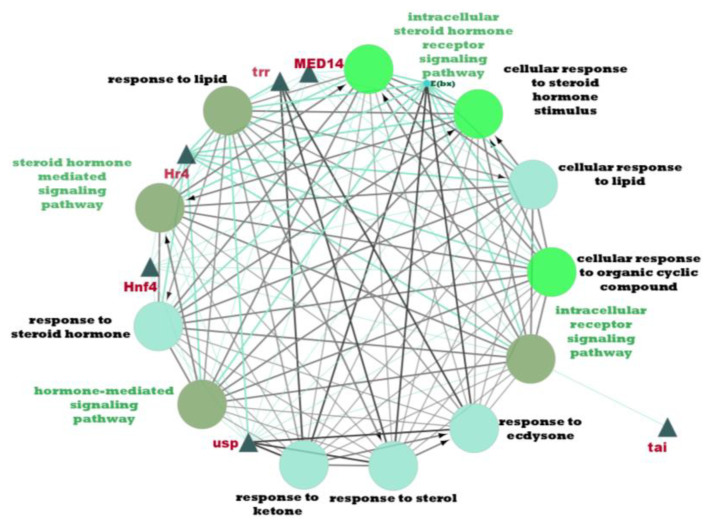
Pathway enrichment analysis by ClueGO/CluePedia in Cytoscape. The triangle and dark turquoise nodes represent the regulatory genes related to the hormone pathways and biological responses related to the steroid hormone pathway, respectively.

From Table 7, a total of 20 potential regulatory genes were identified, i.e., *MTA1-like*, *Nub*, *Grn*, *Usp*, *Hr4*, *Mad*, *Smox*, *Tai*, *Mef2*, *Trx*, *Trr*, *CHES-1-like*, *Skd*, *MED14*, *Cnc*, *Pnt*, *Scr*, *Pygo*, *N*, and *Hnf4*. The following biological processes and pathways were enriched in the 10 most significant clusters, i.e., the steroid hormone-mediated signalling pathway, the intracellular receptor signalling pathway, the hormone-mediated signalling pathway, and the intracellular steroid hormone receptor signalling pathway. Each cluster was characterised based on its biological processes such as the response to ecdysone, response to ketone, cellular response to steroid hormone stimulus, and hormone biosynthesis associated with third instar larvae of *M. plana* (Figure 5).

## 4. Discussion

Palm oil (PO) accounts for one-third of global production of vegetable oil. PO industry is key to global food security and the economic growth of the Southeast Asian countries such as Indonesia, Malaysia, and Thailand (IMT). Within the IMT-triangle countries, 40–45% of the industry is held by independent smallholder farmers who sustain a poor lifestyle with marginal income. In addition, densely populated nations such as India and China, along with other low and middle-income countries (LMIC), engage as ardent PO consumers and major PO importers. Heavy reliance on PO for cooking oil and for various food manufacturing demonstrates the feasible adherence to the four pillars of food security: accessibility, availability, utilisation, and stability. Although the PO industry is negatively associated with sustainability issues, it has been predicted that approximately 7% of annual growth in average yield gain is needed to meet the global demand for vegetable oil by 2050 [49]. Thus, research strategies at tackling yield-limiting factors which include pests and diseases is critical to global PO production and food security [50].

The ongoing *M. plana* infestation in oil palm plantations causes significant yield losses. The current scenario motivates and necessitates the development of strategic and effective pest control management methods [24,51]. However, a lack of comprehensive genetic information on *M. plana* has made it difficult to understand its molecular mechanisms underpinning key biological processes. Since a completed genome sequence of this species is yet to avail, we analysed the *M. plana* transcriptomes corresponding to different developmental stages to unravel new information of its molecular mechanisms, which could potentially provide a basis for further functional analysis studies [23]. We utilised *M. plana* transcriptome data to construct a gene co-expression network on four different pooled developmental stages, i.e., eggs, third instar larvae, pupa, and adult insects. A gene clustering algorithm was applied to identify significant clusters that contain key regulatory genes related to hormone-mediated signalling pathways.

WGCNA generated a comprehensive gene co-expression network comprising 33 co-expressed modules. Only four modules (blue, dark-orange, dark-turquoise, and turquoise) were positively correlated with *M. plana* third instar larvae and therefore, were selected for subsequent analysis (Figure 2B). The modules related to third instar larvae were selected as during the larvae stage, which stretches from the first to the third instar, denoting the ‘active feeder’ status of *M. plana* [24,52]. On the other hand, both moulting and metamorphosis developmental stages are key to growth and development in the general life cycle of insects. Insects under the moulting stage produces new cuticles with subsequent shedding (ecdysis) of the old cuticles [53]. Meanwhile, during metamorphosis, the insects undergo extreme and rapid physical changes, often observed after birth [54]. As a result, the Lepidoptera body plan is rapidly modified during the juvenile to adult transition stage. These modifications are marked as holometabola, whereas a pupal stage is rendered at the interposition between the last larval instar and adult stage. Insect development (moulting) and metamorphosis are controlled by 20-hydroxyecdysone (20E) and juvenile hormone (JH) [55,56,57]. The response triggered by these hormones in each tissue depends on the receptors, the activation or inhibition of precise pathways, and the expression of specific factors that drive the cell-specific response to the stimulus, of which are all primarily governed by the regulatory genes [55].

The combinatorial approach of gene co-expression network and network clustering analysis in *M. plana* identified possible essential regulatory genes related to hormone-mediated pathways, which would possibly remain unresolved using the traditional sequence-based searching method. The application of DPClusO algorithm enabled the discovery and classification of strongly interconnected regions in a large network of core nodes, high connectivity nodes, peripheral nodes, or low connectivity nodes. A similar approach has been used to study various diseases, including inflammatory bowel disease [43] and polycystic ovarian syndrome (PCOS) [58], as well as to identify potential glucosinolate genes in *Arabidopsis thaliana* [41,59,60,61]. DPClusO algorithm creates overlapping clusters depending on the multifunctionality of a gene, fetching greater chances of occurrence in multiple clusters [40,41,62].

Based on the network construction and the clustering analysis, six key regulatory genes were identified: *Hr4*, *Hnf4*, *MED14*, *Usp*, *Tai*, and *Trr*. In insects, the ultraspiracle (*Usp*) regulatory gene is essential in regulating metamorphosis, moulting, and numerous other physiological responses [63]. *Usp* belongs to the nuclear receptor family and is involved in cellular response to lipids, cellular response to steroid hormone stimulus, cellular response to an organic cyclic compound, response to lipid, response to the steroid hormone, response to sterol, response to ecdysone, and response to the ketone. This information suggests the role of up-regulated *Usp* as a key regulator in *M. plana* larvae developmental stage. Meanwhile, the hormone receptor 4 (*Hr4*) regulatory gene, a nuclear receptor (acts both upstream and downstream) of the steroid hormone ecdysone signalling pathway acts as a repressor in the production of ecdysone in the prothoracic gland. Other than that, in *Drosophila*, ecdysone was reported to induce *Hr4* for subsequent transduction of hormonal effects to other body tissues, with the salivary gland and body fat tissues, among them [64].

Both *Usp* and *Hr4* genes are enriched in four different hormone-mediated pathways: (i) hormone-mediated signalling pathway, (ii) intracellular steroid-hormone receptor signalling pathway, (iii) steroid-hormone mediated signalling pathway, and (iv) intracellular receptor signalling pathway. Furthermore, *Hr4* is involved in various biological processes: i.e., cellular response to lipids, cellular response to steroid hormone stimulus, cellular response to an organic cyclic compound, lipids, and responses to a steroid hormone. The activity or inactivity of prothoracicotropic hormone (PTTH) signalling determines whether DHR4 suppresses ecdysone pulses in *Drosophila melanogaster*. In the presence of PTTH signalling, DHR4 is repressed from the nucleus either via shuttle movement to the cytoplasm or through protein degradation. As a result, ecdysone biosynthesis takes place. Under a PTTH absent state, DHR4 remains in the nucleus and represses *Cyp6t3*, along with other genes responsible for ecdysone production; consequently, it is responsible for lowering ecdysone titers. Hence, in *Drosophila* development, DHR4 is a critical regulatory gene that controls the timing of hormone pulses [65].

The Hepatocyte nuclear factor 4 (*Hnf4*) regulatory gene is enriched in three hormone-mediated pathways: (i) hormone-mediated signalling pathway, (ii) steroid hormone-mediated signalling pathway, and (iii) intracellular receptor signalling pathway. Similar to *Hr4*, the *Hnf4* gene is involved in biological processes. *Hnf4* also belongs to the nuclear receptors family that encodes for lipid mobilisation and fatty acid beta-oxidation regulating protein at the larval stages. At the onset of *D. melanogaster* adulthood, the expression of *Hnf4* increases in favour of glucose-stimulated ILP secretion from the insulin-producing cells, ultimately to maintain glucose homeostasis and to support mitochondrial function [66]. The Mediator complex subunit 14 (*MED14)* regulatory gene is enriched in similar biological processes as with *Hr4* and *Hnf4* genes. In addition, *MED14* is enriched in all hormone-mediated pathways. *MED14* acts as a co-activator that regulates the transcription of RNA-polymerase-dependent genes and mediates as a bridge to convey information from gene-specific regulatory proteins to the basal RNA polymerase II transcription machinery in *D. melanogaster* [55]. However, there is limited knowledge of these genes *Hr4*, *Hnf4*, and *MED14* on other lepidopteran species. However, in the development of *M. plana* larvae, the expression of these three genes was downregulated.

The *Tai* regulatory gene is also identified as one of the key regulatory genes. The expression of *Tai* in the *M. plana* third instar larvae stage was upregulated. Taiman, also known as *Tai*, is a steroid receptor, a co-activator of 20-hydroxyecdysone (20E) receptor. In several insect species, *Tai* forms a heterodimer complex with the ecdysone receptor (*EcR*) and ultraspiracle (*Usp*) to control the differentiation of early germline cells and facilitate the migration of specific follicle cells and border cells in ovaries. In addition, *Tai* binds to methoprene-tolerant to form a heterodimeric complex, mediating juvenile hormone (JH) signalling to regulate larval development and to prevent premature metamorphosis [67]. The *Tai* regulatory gene is enriched only in the intracellular receptor signalling pathway and has no interactions between the biological responses.

Likewise, the trithorax-related, *Trr* regulatory gene identified in this study was not involved in any hormone-mediated signalling pathways. However, it was enriched in several biological responses, such as the response to ecdysone, response to sterol, response to ketone, and response to lipid. In *D. melanogaster*, *trr* activates two-target genes, *hedgehog*, *hh* and *BR-C*, through the interactions between *EcR* and *Usp* that binds to ecdysone [68]. However, the expression of *Trr* was upregulated in *M. plana’s* third instar larvae stage.

In insects, ecdysone is classified as a steroid hormone, and plays overlapping functions in both the general hormone and steroid-hormone mediating signalling pathways. These signalling pathways are mostly regulated by nuclear [65,66] receptors. The regulatory genes identified in *M. plana* are crucial as the moulting process is active throughout the growth of instars. To the best of our knowledge, this is the first report on the discovery of regulatory genes in *M. plana*. The generalisability of the findings presented herein may be limited as it only focused on *M. plana* and the transcriptomes corresponding to specific life stages. The results are specific to *M. plana* and thus, it remains uncertain whether these findings can be referred to either other bagworm species or different developmental stages from which has been reported in *M. plana*. Even though the study has identified potential key regulatory genes associated with *M. plana* infestation, the effectiveness of these targets in controlling the pest has not been experimentally validated. Therefore, further research, such as RNA interference (RNAi) or overexpression studies, are necessary to validate the potential utility of these targets in controlling *M. plana* infestation. Validation experiments are crucial to confirm the function of these targets before they can be used in pesticide development or other control strategies. In addition, RNAi or overexpression studies can provide insights into the specific functional roles of these genes in regulating hormone pathways and other physiological processes in *M. plana*.

The identified regulatory genes upon validation can be considered potential targets in RNAi-based pest control management, and they can be developed as a promising tool in integrated pest management [69]. RNAi mechanisms are increasingly used to silence the essential genes in pest insects, which illustrates a promising strategy for the integrated pest management (IPM) approach [33]. Furthermore, numerous studies have shown the feasibility of regulatory genes as hormones receptor and excellent targets in insecticide development with pest specificity [70]. For instance, the occasion of the ecdysone receptors EcR (*SaEcR)* and USP (*SaUsp*) of grain aphid *Sitobion avenae* silenced using RNAi technology has been shown to reduce fecundity and survival, and as a result, both genes have been successfully used as RNAi targets for wheat aphid control [71].

Although the study successfully identified key regulatory genes involved in *M. plana* hormone pathways during the third instar larvae stage, it did not explore all plausible genetic factors that contribute to *M. plana* infestation. Nevertheless, focusing on the third instar larvae stage was a relevant and effective strategy since this stage is critical in *M. plana* development as is associated with feeding behaviour and deemed the highest risk of infestation to oil palms. In support of this strategy, a study by Xu et al. (2020) identified key genes associated with overwintering in the larvae of *Anoplophora glabripennis* [72]. Clustering analysis is a commonly used method to identify potential regulatory genes in a network. Nevertheless, it is not infallible and may generate false positives due to various factors such as random variation and noise. To address this limitation, the current study employed Fisher’s exact test and ROC analysis to evaluate the significance and accuracy of clustering results. In addition to clustering analysis, the study employed pathway enrichment analysis in Cytoscape via the ClueGo/CluePedia plug-in to identify potential regulatory genes. These genes were selected based on their involvement in the hormone pathways. This knowledge-based approach could minimise the existence of false positives present in this study.

## 5. Conclusions

The combinatorial approach of gene co-expression and network clustering analysis has revealed key regulatory genes (*Hnf4*, *Hr4*, *MED14*, *Usp*, *Tai*, and *Trr*) involved in the hormone biosynthesis pathway during the third instar of M. plana larvae. Validation experiments are required to confirm their potentials as targets in RNAi, gene silencing technology and subsequent up-stream application in the development of species-specific biopesticides.

## Figures and Tables

**Table 1:** The contingency table prepared in this study calculates the known regulatory gene enrichment clusters.

	Regulatory Genes	Non-Regulatory Genes
In cluster	a	b
Not in cluster	c	d
Total	a + c	b + d

Note: a, b, c, and d refer to the independent total number of genes in the clusters.

**Table 2:** Descriptive information of *M. plana* third instar larvae modules, extracted from a weighted gene co-expression network analysis (WGCNA) of transcriptomes datasets. Module columns represented by different colour codes represent a unique module: blue, dark-orange, dark-turquoise, and turquoise.

Modules	Number of Co-Expressed Genes	Number of Genes with MM ≥ 0.80
Blue	636	576
Dark-orange	49	25
Dark-turquoise	58	71
Turquoise	2522	1918

**Table 3:** Descriptive statistics of hub gene annotations present in unique modules of *M. plana* third instar larvae. Module column represented by different colour codes represents a unique module: blue, dark-orange, dark-turquoise and turquoise.

Modules	Number of Hub Genes	Number of Genes without Annotation	Number of Genes Annotated by ARGOT	Genes without Annotation by ARGOT
Blue	576	216	149	67
Dark-orange	25	5	5	0
Dark-turquoise	71	14	9	5
Turquoise	1918	857	624	233
Total	2590	1092	787	305

**Table 4:** Top 20 clusters with corresponding enriched representative terms (one per cluster). “Count” denotes the number of genes in the user-provided lists with membership in the given ontology term. “Log10(q)” represents the multi-test adjusted *p*-value in log base 10.

GO	Category	Description	Count	Log10(q)
GO:0035295	GO BP	tube development	67	−8.89
GO:0042692	GO BP	muscle cell differentiation	19	−5.68
GO:0060541	GO BP	respiratory system development	30	−5.68
GO:0003002	GO BP	regionalisation	45	−5.38
GO:0032989	GO BP	cellular component morphogenesis	47	−5.35
GO:0002376	GO BP	immune system process	32	−4.93
GO:0097305	GO BP	response to alcohol	18	−4.22
GO:0048565	GO BP	digestive tract development	17	−3.97
GO:0040008	GO BP	regulation of growth	30	−3.89
GO:0042592	GO BP	homeostatic process	38	−3.89
GO:0007166	GO BP	cell surface receptor signalling pathway	29	−3.76
GO:0007447	GO BP	imaginal disc pattern formation	16	−3.48
GO:0002164	GO BP	larval development	21	−3.48
GO:0044281	GO BP	small molecule metabolic process	52	−3.45
GO:0022408	GO BP	negative regulation of cell-cell adhesion	6	−3.23
R-DME−9006931	Reactome Gene Sets	signalling by nuclear receptors	15	−3.16
GO:0051604	GO BP	protein maturation	16	−3.16
GO:0012501	GO BP	programmed cell death	18	−2.95
GO:0008037	GO BP	cell recognition	15	−2.86
GO:0010876	GO BP	lipid localisation	14	−2.81

GO: Gene Ontology; BP: Biological Processes.

**Table 5:** Cluster properties of different input densities using DPClusO algorithm.

Density	Number of Clusters	Maximum Size of Clusters
0.5	193	1071
0.6	227	991
0.7	275	1136
0.8	291	1074
0.9	307	913

**Table 6:** The area under each density curve (AUC) from the Receiver Operating Characteristics (ROC) analysis.

Density	Area under the Curve (AUC)
0.5	0.913
0.6	0.950
0.7	0.824
0.8	0.947
0.9	0.934

**Table 7:** List of potential regulatory genes from significant clusters.

Cluster Number	Cluster Size	Regulatory Genes	Cluster	*p*-Value
Cluster 3	15	*MTA1-like*, *Nub, Grn*, *Usp*, *Hr4, Mad, Smox*, *Tai*, *Mef2*	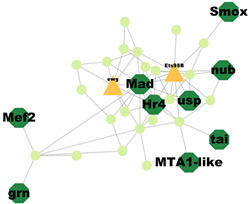	3.52 × 10^−7^
Cluster 7	12	*Trx*, *Trr*, *Usp*, *Hr4*	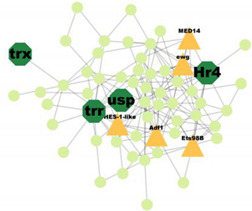	1.52 × 10^−3^
Cluster 8	12	*Mad, Smox, Ches-1-like, Usp, Hr4, Tai, Mef2*	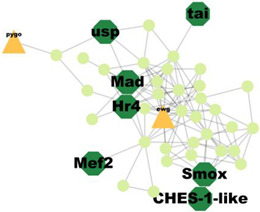	1.25 × 10^−5^
Cluster 9	12	*Skd*, *MED14*, *Usp*, *Hr4*, *Mad*, *Smox*, *Tai*, *Mef2*	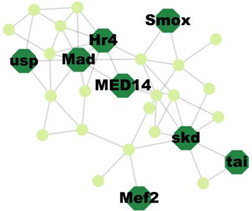	5.83 × 10^−7^
Cluster 11	12	*Cnc*, *Mad*, *Smox*, *Usp*, *Hr4*, *Tai*, *Mef2*	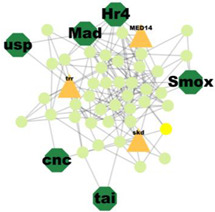	1.25 × 10^−5^
Cluster 12	11	*Usp, Hr4, Mad, Smox, Tai, Mef2, Grn*	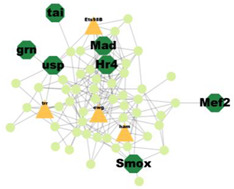	5.36 × 10^−6^
Cluster 14	11	*Pnt, Mef2, Scr, Mad, Smox, Usp, Hr4, Tai*	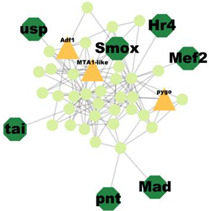	2.07 × 10^−7^
Cluster 24	8	*Pygo, N, Trr*	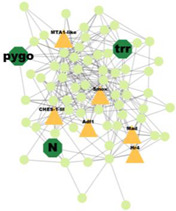	2.613 × 10^−2^
Cluster 47	5	*Hr4, Hnf4, Usp*	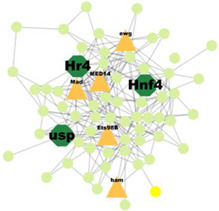	5.652 × 10^−3^
Cluster 53	5	*Smox, Hnf4, Mad*	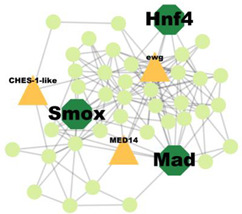	5.625 × 10^−3^

Octagon Node: Potential regulatory genes.

## Data Availability

The data used in this analysis were obtained upon request from the cited publisher. The data are available upon request from the corresponding author.

## Supplementary Materials

The following supporting information can be downloaded at: https://www.mdpi.com/article/10.3390/insects14060503/s1, Figure S1: Hierarchical clustering to detect sample outliers. There were no outliers detected in the samples. Figure S2: Analysis of the scale-free fit and mean connectivity for soft-thresholding powers (β). Figure S3: Bar chart representing the module’s colours generated from the network. Figure S4: Heatmap to show the relationship between the modules and the different developmental stages of *Metisa plana*. Each row corresponds to a module eigengene, and each column corresponds to the developmental stages of the *Metisa plana*. Each cell contains the corresponding correlations. Table S1: Number of Genes in each module. Table S2: List of hub genes with module membership of ≥0.80 associated with the modules related to larvae of *Metisa plana*.

## References

[1] Murphy D.J., Goggin K., Paterson R.R.M. (2021). Oil palm in the 2020s and beyond: Challenges and solutions. CABI Agric. Biosci..

[2] Carter C., Finley W., Fry J., Jackson D., Willis L. (2007). Palm oil markets and future supply. Eur. J. Lipid Sci. Technol..

[3] Shevade V.S., Loboda T.V. (2019). Oil palm plantations in Peninsular Malaysia: Determinants and constraints on expansion. PLoS ONE.

[4] Wicke B., Sikkema R., Dornburg V., Faaij A. (2011). Exploring land use changes and the role of palm oil production in Indonesia and Malaysia. Land Use Policy.

[5] Nambiappan B. (2018). Malaysia: 100 years of resilient palm oil economic performance. J. Oil Palm Res..

[6] Koh L.P., Wilcove D.S. (2007). Cashing in palm oil for conservation. Nature.

[7] Foster W.A., Snaddon J.L., Turner E., Fayle T., Cockerill T.D., Ellwood M.D.F., Broad G.R., Chung A.Y.C., Eggleton P., Khen C.V. (2011). Establishing the evidence base for maintaining biodiversity and ecosystem function in the oil palm landscapes of South East Asia. Philos. Trans. R. Soc. B Biol. Sci..

[8] Jamian S., Norhisham A., Ghazali A., Zakaria A., Azhar B. (2016). Impacts of 2 species of predatory Reduviidae on bagworms in oil palm plantations. Insect Sci..

[9] Thaer S., Kassim F.A., Hasbullah N.A., Al-obaidi J.R. (2021). Evaluation of Bagworm, Metisa plana (*Lepidoptera*: *Psychidae*) Infestation and Beneficial Parasitoid in an Oil Palm Plantation, Perak, Malaysia. J. Sci. Math. Lett..

[10] Halim M., Muhaimin A.M.D., Zulaikha S.A.S., Atikah A.R.N., Masri M.M.M., Yaakop S. (2017). Evaluation of infestation in parasitoids on Metisa plana Walker (*Lepidoptera*: *Psychidae*) in three oil palm plantations in peninsular Malaysia. Serangga.

[11] (2022). Production of Crude Palm Oil in Malaysia 2012–2021. https://www.statista.com/statistics/489441/palm-oil-consumption-malaysia/.

[12] Tey C.-C., Loong C.-Y. (2012). Understanding Pest Biology and Behaviour for Effective Control of Oil Palm Bagworms. Plant. Kuala Lumpur..

[13] Pradana M.G., Priwiratama H., Rozziansha T.A.P., Prasetyo A.E., Susanto A. (2022). Field evaluation of Bacillus thuringiensis product to control Metisa plana bagworm in oil palm plantation. IOP Conf. Ser. Earth Environ. Sci..

[14] Kamarudin N., Ali S.R.A., Masri M.M.M., Ahmad M.N., Manan C.A.H.C., Kamarudin N. (2017). Controlling Metisa plana Walker (*Lepidoptera*: *Psychidae*) outbreak using bacillus thuringiensis at an oil palm plantation in slim river, perak, malaysia. J. Oil Palm Res..

[15] Kok C.C., Eng O.K., Razak A.R., Arshad A.M. (2011). Microstructure and life cycle of Metisa plana Walker (*Lepidoptera*: *Psychidae*). J. Sustain. Sci. Manag..

[16] Salim H., Rawi C.S., Ahmad A.H., Al-Shami S.A. (2015). Efficacy of Insecticide and Bioinsecticide Ground Sprays to Control Metisa plana Walker (*Lepidoptera*: *Psychidae*) in Oil Palm Plantations, Malaysia. Trop. Life Sci. Res..

[17] Mirth C.K., Tang H.Y., Makohon-Moore S.C., Salhadar S., Gokhale R.H., Warner R.D., Koyama T., Riddiford L.M., Shingleton A.W. (2014). Juvenile hormone regulates body size and perturbs insulin signaling in *Drosophila*. Proc. Natl. Acad. Sci. USA.

[18] Semsey S., Adhya S. (2013). Regulatory Genes. Brenner’s Encyclopedia of Genetics.

[19] Uryu O., Ou Q., Komura-Kawa T., Kamiyama T., Iga M., Syrzycka M., Hirota K., Kataoka H., Honda B.M., King-Jones K. (2018). Cooperative Control of Ecdysone Biosynthesis in *Drosophila* by Transcription Factors Séance, Ouija Board, and Molting Defective. Genetics.

[20] Guo Z., Qin J., Zhou X., Zhang Y. (2018). Insect Transcription Factors: A Landscape of Their Structures and Biological Functions in Drosophila and beyond. Int. J. Mol. Sci..

[21] (2023). Britannica, “Gene,” Encyclopedia Britannica. https://www.britannica.com/science/gene.

[22] Zeng B., Huang Y., Xu J., Shiotsuki T., Bai H., Palli S.R., Huang Y., Tan A. (2017). The FOXO transcription factor controls insect growth and development by regulating juvenile hormone degradation in the silkworm, Bombyx mori. J. Biol. Chem..

[23] Rahmat N.L., Zifruddin A.N., Abidin C.M.R.Z., Muhammad N.-A.N., Hassan M. (2020). The Developmental Transcriptome of Bagworm, *Metisa plana* (*Lepidoptera*: *Psychidae*) and Insights into Chitin Biosynthesis Genes. Genes.

[24] Zainuddin N., Maidin M.S.T., Kamarudin N., Napiah N.R.A.M.A., Keni M.F., Masri M.M.M. (2023). De novo transcriptome analysis of bagworm Metisa plana from highly infested oil palm estate in Perak revealed detoxification genes and potential insecticide targets. J. Asia-Pac. Èntomol..

[25] Langfelder P., Horvath S. (2008). WGCNA: An R package for weighted correlation network analysis. BMC Bioinform..

[26] Bakhtiarizadeh M.R., Hosseinpour B., Shahhoseini M., Korte A., Gifani P. (2018). Weighted Gene Co-expression Network Analysis of Endometriosis and Identification of Functional Modules Associated With Its Main Hallmarks. Front. Genet..

[27] Bakhtiarizadeh M.R., Mirzaei S., Norouzi M., Sheybani N., Sadi M.S.V. (2020). Identification of Gene Modules and Hub Genes Involved in Mastitis Development Using a Systems Biology Approach. Front. Genet..

[28] Fan X.-B., Pang R., Li W.-X., Ojha A., Li D., Zhang W.-Q. (2020). An Overview of Embryogenesis: External Morphology and Transcriptome Profiling in the Hemipteran Insect Nilaparvata lugens. Front. Physiol..

[29] Ding W.-F., Ling X.-F., Lu Q., Wang W.-W., Zhang X., Feng Y., Chen X.-M., Chen H. (2022). Identification of the Key Pathways and Genes Involved in the Wax Biosynthesis of the Chinese White Wax Scale Insect (*Ericerus pela* Chavannes) by Integrated Weighted Gene Coexpression Network Analysis. Genes.

[30] Katoch R., Sethi A., Thakur N., Murdock L.L. (2013). RNAi for Insect Control: Current Perspective and Future Challenges. Appl. Biochem. Biotechnol..

[31] Mehlhorn S., Hunnekuhl V.S., Geibel S., Nauen R., Bucher G. (2021). Establishing RNAi for basic research and pest control and identification of the most efficient target genes for pest control: A brief guide. Front. Zool..

[32] Whyard S., Singh A.D., Wong S. (2009). Ingested double-stranded RNAs can act as species-specific insecticides. Insect Biochem. Mol. Biol..

[33] Christiaens O., Whyard S., Vélez A.M., Smagghe G. (2020). Double-Stranded RNA Technology to Control Insect Pests: Current Status and Challenges. Front. Plant Sci..

[34] Zhu F., Xu J., Palli R., Ferguson J., Palli S.R. (2010). Ingested RNA interference for managing the populations of the Colorado potato beetle, *Leptinotarsa decemlineata*. Pest Manag. Sci..

[35] Zhang Z., Ma Y., Ma X., Hu H., Wang D., Song X., Ren X., Ma Y. (2021). Combined Transcriptomic Analysis and RNA Interference Reveal the Effects of Methoxyfenozide on Ecdysone Signaling Pathway of *Spodoptera exigua*. Int. J. Mol. Sci..

[36] Love M.I., Huber W., Anders S. (2014). Moderated estimation of fold change and dispersion for RNA-seq data with DESeq2. Genome Biol..

[37] Falda M., Toppo S., Pescarolo A., Lavezzo E., Di Camillo B., Facchinetti A., Cilia E., Velasco R., Fontana P. (2012). Argot2: A large scale function prediction tool relying on semantic similarity of weighted Gene Ontology terms. BMC Bioinform..

[38] Zhou Y., Zhou B., Pache L., Chang M., Khodabakhshi A.H., Tanaseichuk O., Benner C., Chanda S.K. (2019). Metascape provides a biologist-oriented resource for the analysis of systems-level datasets. Nat. Commun..

[39] Shannon P., Markiel A., Ozier O., Baliga N.S., Wang J.T., Ramage D., Amin N., Schwikowski B., Ideker T. (2003). Cytoscape: A software environment for integrated models of Biomolecular Interaction Networks. Genome Res..

[40] Karim M.B., Wakamatsu N., Altaf-Ul-Amin M. (2017). DPClusOST: A Software Tool for General Purpose Graph Clustering. J. Comput. Aided Chem..

[41] Harun S., Afiqah-Aleng N., Karim M.B., Amin A.U., Kanaya S., Mohamed-Hussein Z.-A. (2021). Potential *Arabidopsis thaliana* glucosinolate genes identified from the co-expression modules using graph clustering approach. PeerJ.

[42] Karim M.B.B., Huang M., Ono N., Kanaya S., Amin A.-U. (2019). BiClusO: A Novel Biclustering Approach and Its Application to Species-VOC Relational Data. IEEE/ACM Trans. Comput. Biol. Bioinform..

[43] Eguchi R., Karim M.B., Hu P., Sato T., Ono N., Kanaya S., Amin A.U. (2018). An integrative network-based approach to identify novel disease genes and pathways: A case study in the context of inflammatory bowel disease. BMC Bioinform..

[44] Fisher R.A. (1992). Statistical Methods for Research Workers.

[45] Fisher R.A. (1922). On the Interpretation of χ^2^ from Contingency Tables, and the Calculation of P. J. R. Stat. Soc..

[46] Metz C.E. (1978). Basic principles of ROC analysis. Semin Nucl. Med..

[47] Sing T., Sander O., Beerenwinkel N., Lengauer T. (2005). ROCR: Visualizing classifier performance in R. Bioinformatics.

[48] Bindea G., Mlecnik B., Hackl H., Charoentong P., Tosolini M., Kirilovsky A., Fridman W.-H., Pagès F., Trajanoski Z., Galon J. (2009). ClueGO: A Cytoscape plug-in to decipher functionally grouped gene ontology and pathway annotation networks. Bioinformatics.

[49] Purnomo H., Okarda B., Dermawan A., Ilham Q.P., Pacheco P., Nurfatriani F., Suhendang E. (2020). Reconciling oil palm economic development and environmental conservation in Indonesia: A value chain dynamic approach. For. Policy Econ..

[50] Jackson T.A., Crawford J.W., Traeholt C., Sanders T.A.B. (2019). Learning to love the world’s most hated crop—Review Articles. J. Oil Palm Res..

[51] Ting G.A., Jie A.C., Abidin M.R.Z., Hamid N.H., Salim H. (2021). 16S rRNA Amplicon Sequencing of Bagworm Metisa plana Walker (*Lepidoptera*: *Psychidae*). BioRxiv.

[52] Kok C.C., Eng O.K., Razak A.R., Arshad A.M., Marcon P.G. (2012). Susceptibility of Bagworm Metisa plana (*Lepidoptera*: *Psychidae*) to chlorantraniliprole. Pertanika J. Trop Agric. Sci..

[53] Riddiford L.M. (2009). Molting. Encyclopedia of Insects.

[54] Kaleka A.S., Kaur N., Bali G.K. (2019). Larval Development and Molting. Edible Insects.

[55] Tettamanti G., Casartelli M. (2019). Cell death during complete metamorphosis. Philos. Trans. R. Soc. B Biol. Sci..

[56] Yamanaka N., Rewitz K.F., O’Connor M.B. (2013). Ecdysone Control of Developmental Transitions: Lessons from *Drosophila* Research. Annu. Rev. Èntomol..

[57] Jindra M., Palli S.R., Riddiford L.M. (2013). The Juvenile Hormone Signaling Pathway in Insect Development. Annu. Rev. Èntomol..

[58] Afiqah-Aleng N., Altaf-Ul-Amin M., Kanaya S., Mohamed-Hussein Z.-A. (2019). Graph cluster approach in identifying novel proteins and significant pathways involved in polycystic ovary syndrome. Reprod. Biomed. Online.

[59] Harun S., Abdullah-Zawawi M.-R., Goh H.-H., Mohamed-Hussein Z.-A. (2020). A Comprehensive Gene Inventory for Glucosinolate Biosynthetic Pathway in *Arabidopsis thaliana*. J. Agric. Food Chem..

[60] Harun S., Rohani E.R., Ohme-Takagi M., Goh H.-H., Mohamed-Hussein Z.-A. (2021). ADAP is a possible negative regulator of glucosinolate biosynthesis in Arabidopsis thaliana based on clustering and gene expression analyses. J. Plant Res..

[61] Harun S., Abdullah-Zawawi M.-R., A-Rahman M.R.A., Muhammad N.A.N., Mohamed-Hussein Z.-A. (2019). SuCComBase: A manually curated repository of plant sulfur-containing compounds. Database.

[62] Amin A.U., Wada M., Kanaya S. (2012). Partitioning a PPI Network into Overlapping Modules Constrained by High-Density and Periphery Tracking. ISRN Biomath..

[63] Jones G., Teal P., Henrich V.C., Krzywonos A., Sapa A., Wozniak M., Smolka J., Jones D. (2013). Ligand binding pocket function of Drosophila USP is necessary for metamorphosis. Gen. Comp. Endocrinol..

[64] King-Jones K., Charles J.-P., Lam G., Thummel C.S. (2005). The Ecdysone-Induced DHR4 Orphan Nuclear Receptor Coordinates Growth and Maturation in Drosophila. Cell.

[65] Ou Q., Magico A., King-Jones K. (2011). Nuclear Receptor DHR4 Controls the Timing of Steroid Hormone Pulses During Drosophila Development. PLoS Biol..

[66] Barry W.E., Thummel C.S. (2016). The Drosophila HNF4 nuclear receptor promotes glucose-stimulated insulin secretion and mitochondrial function in adults. eLife.

[67] Xu Q.-Y., Du J.-L., Mu L.-L., Guo W.-C., Li G.-Q. (2019). Importance of Taiman in Larval-Pupal Transition in Leptinotarsa decemlineata. Front. Physiol..

[68] Johnston D.M., Sedkov Y., Petruk S., Riley K.M., Fujioka M., Jaynes J.B., Mazo A. (2011). Ecdysone- and NO-Mediated Gene Regulation by Competing EcR/Usp and E75A Nuclear Receptors during Drosophila Development. Mol. Cell.

[69] Yu R., Xu X., Liang Y., Tian H., Pan Z., Jin S., Wang N., Zhang W. (2014). The Insect Ecdysone Receptor is a Good Potential Target for RNAi-based Pest Control. Int. J. Biol. Sci..

[70] Masterson M., Bittar R., Chu H., Yamanaka N., Haga-Yamanaka S. (2022). Rapid Assessment of Insect Steroid Hormone Entry Into Cultured Cells. Front. Physiol..

[71] Yan T., Chen H., Sun Y., Yu X., Xia L. (2016). RNA Interference of the Ecdysone Receptor Genes EcR and USP in Grain Aphid (*Sitobion avenae* F.) Affects Its Survival and Fecundity upon Feeding on Wheat Plants. Int. J. Mol. Sci..

[72] Xu Y., Li Y., Wang Q., Zheng C., Zhao D., Shi F., Liu X., Tao J., Zong S. (2020). Identification of key genes associated with overwintering in *Anoplophora glabripennis* larva using gene co-expression network analysis. Pest Manag. Sci..

