# Supplementary material for: Gene Co-Expression Network Analysis Reveals Key Regulatory Genes in Metisa plana Hormone Pathways
Source: Insects. 2023 May 30;14(6):503. doi: 10.3390/insects14060503 (PMC10299145; doi:10.3390/insects14060503)
Supplement: Supplementary file 1 [file insects-14-00503-s001.zip › Supplementary Material-27-03-2023.pdf]

Supplementary Materials

Gene Co-expression Network Analysis reveals Key Regulatory Genes of Insect Hormone Pathways in *Metisa plana*

Vinothienii Vengatharajuloo[1], Hoe-Han Goh[1], Maizom Hassan[1], Nor Afiqah Aleng[2], Nisha Govender[1]

Sarahani Harun[1], Zeti-Azura Mohamed-Husein[1,3]

Figures and Table Legends

Figure S1: Hierarchical clustering to detect sample outliers. There were no outliers detected in the samples.

Figure S2: Analysis of the scale-free fit and mean connectivity for soft-thresholding powers (β).

Figure S3: Bar chart representing the module's colours generated from the network.

Figure S4: Heatmap to show the relationship between the modules and the different developmental stages of *Metisa plana*. Each row corresponds to a module eigengene, and each column corresponds to the developmental stages of the *Metisa plana*. Each cell contains the corresponding correlations.

Table S1: Number of Genes in each module.

Table S2: List of hub genes with module membership of ≥ 0.80 associated with the modules related to larvae of *Metisa plana*.

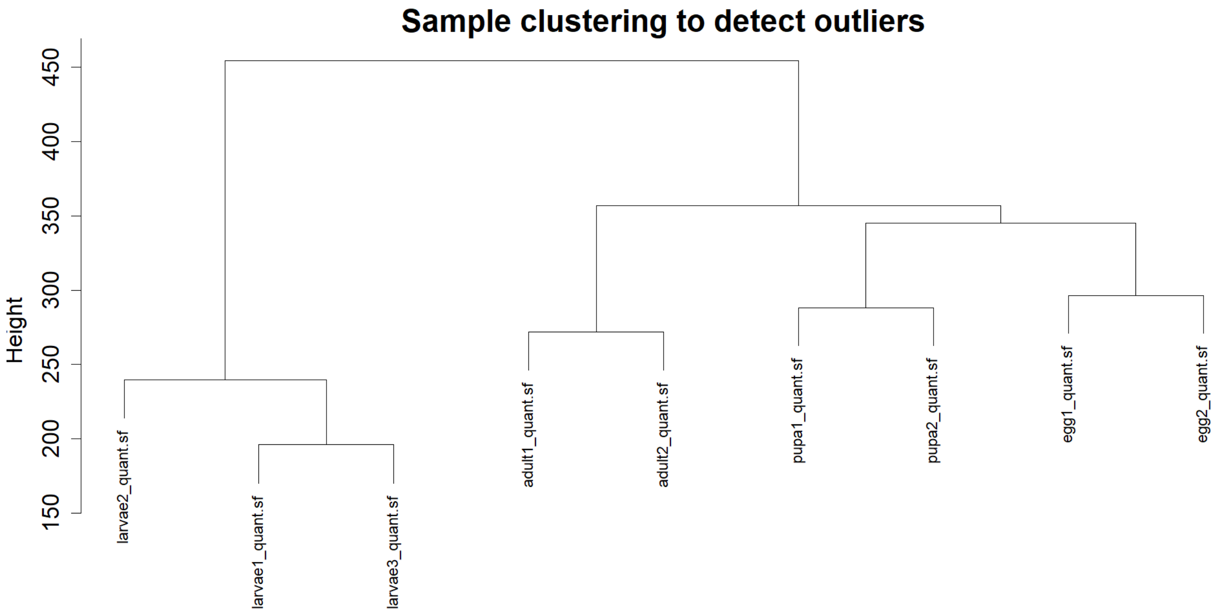

Figure S1. Hierarchical clustering to detect sample outliers. There were no outliers detected in the samples.

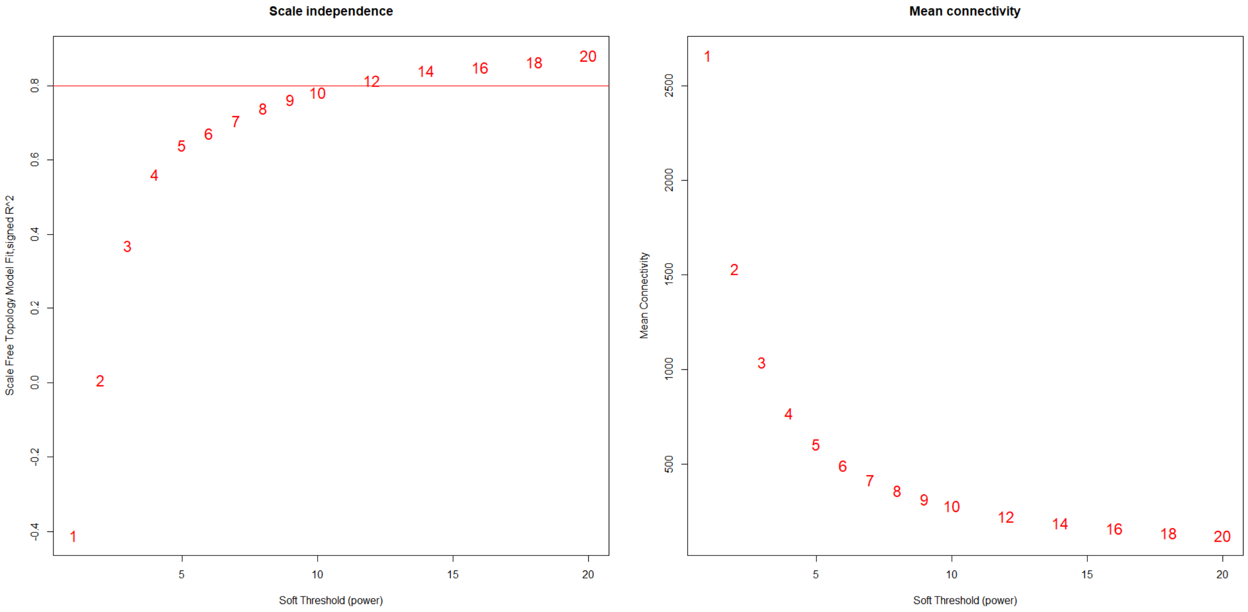

Figure S2. Analysis of the scale-free fit and mean connectivity for various soft-thresholding powers (β).

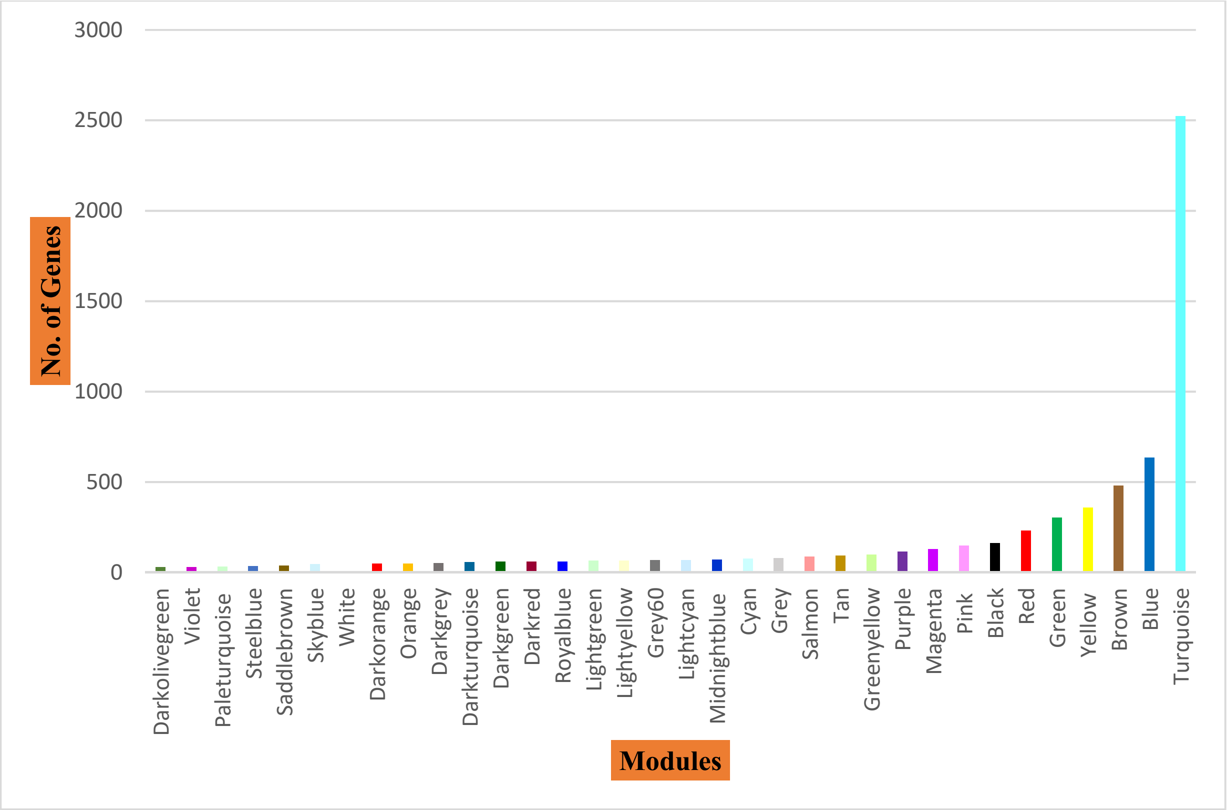

Figure S3. Bar chart representing the module's colours generated from the network.

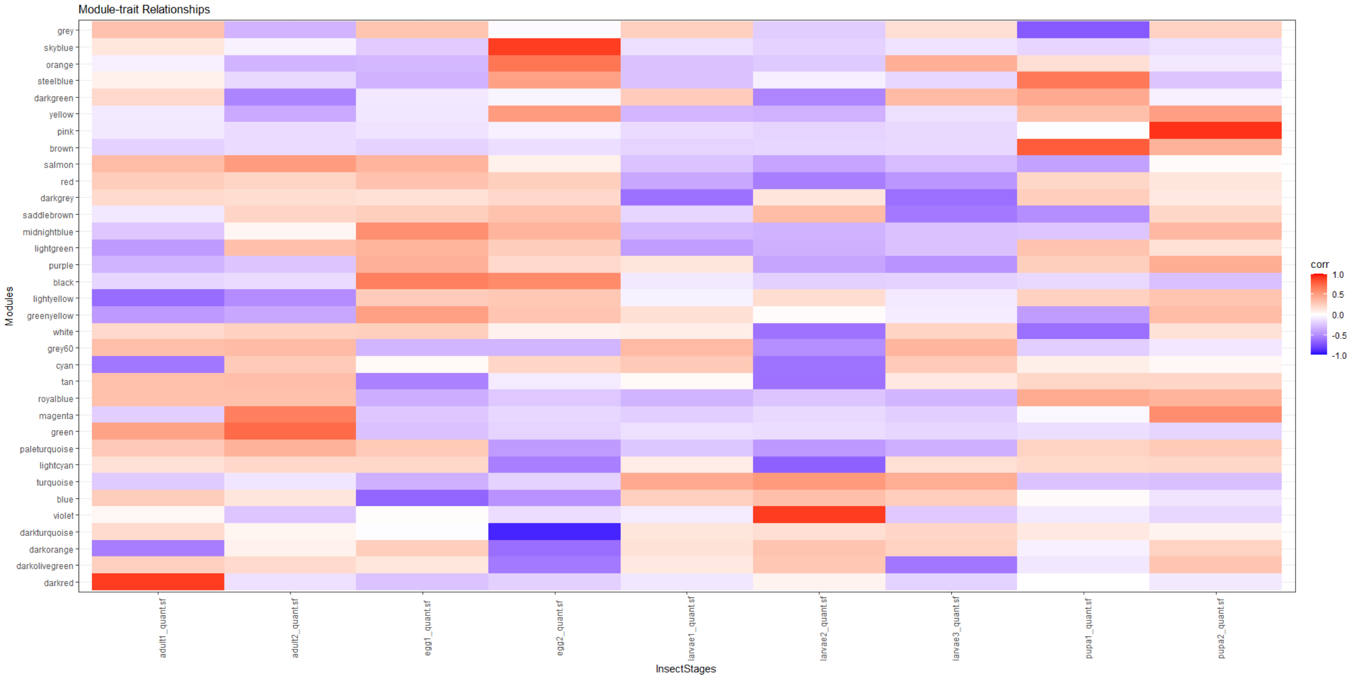

Figure S4: Heatmap to show the relationship between the modules and the different developmental stages of *Metisa plana*. Each row corresponds to a module eigengene, and each column corresponds to the developmental stages of the *Metisa plana*. Each cell contains the corresponding correlations.
